# Overexpression of the *Brassica rapa* bZIP Transcription Factor, *BrbZIP-S*, Increases the Stress Tolerance in *Nicotiana benthamiana*

**DOI:** 10.3390/biology12040517

**Published:** 2023-03-29

**Authors:** Seung Hee Eom, Heung Bin Lim, Tae Kyung Hyun

**Affiliations:** Department of Industrial Plant Science and Technology, College of Agriculture, Life and Environment Sciences, Chungbuk National University, Cheongju 28644, Republic of Korea; eom0214@naver.com

**Keywords:** heat stress, proline, S_1_-basic region-leucine zipper, senescence, sugar metabolism

## Abstract

**Simple Summary:**

Energy homeostasis plays a crucial role in regulating plant defense responses. In this study, we characterized BrbZIP-S (S_1_-bZIP from *Brassica rapa*) as a key modulator of energy metabolism, including sugar and proline metabolism. In addition, plants overexpressing *BrbZIP-S* exhibited increased tolerance to darkness and heat stress, suggesting that BrbZIP-S regulates plant stress responses through a complex network mediated by abscisic acid, sugar, and proline.

**Abstract:**

In higher plants, S_1_-basic region-leucine zipper (S_1_-bZIP) transcription factors fulfill crucial roles in the physiological homeostasis of carbon and amino acid metabolisms and stress responses. However, very little is known about the physiological role of S_1_-bZIP in cruciferous vegetables. Here, we analyzed the physiological function of S_1_-bZIP from *Brassica rapa* (BrbZIP-S) in modulating proline and sugar metabolism. Overexpression of *BrbZIP-S* in *Nicotiana benthamiana* resulted in delayed chlorophyll degradation during the response to dark conditions. Under heat stress or recovery conditions, the transgenic lines exhibited a lower accumulation of H_2_O_2_, malondialdehyde, and protein carbonyls compared to the levels in transgenic control plants. These results strongly indicate that BrbZIP-S regulates plant tolerance against dark and heat stress. We propose that BrbZIP-S is a modulator of proline and sugar metabolism, which are required for energy homeostasis in response to environmental stress conditions.

## 1. Introduction

Energy homeostasis in plant cells is regulated by diverse mechanisms concurring with the plant’s response to environmental conditions. Energy is available in the form of carbon fixed through photosynthesis, whereas various environmental stresses affect photosynthetic carbon metabolism by affecting photosynthesis, carbon allocation, respiration, and so on [1,2]. Thus, energy management systems, including catabolic processes and anabolism repression are required for plant growth and development under energy-deprived conditions [3]. As an energy sensor, sucrose non-fermenting-1-related kinases 1 (SnRK1) assists in transcriptional and metabolic reprogramming, which regulates the carbohydrate metabolism and energy balance under low-energy stress [4,5]. Importantly, members of the basic region-leucine zipper (bZIP) transcription factor family have been found to be SnRK1-downstream mediators, which regulate the transcription of genes involved in various catabolic pathways providing alternative sources of energy and metabolites [3].

In the Arabidopsis genome, the bZIP family comprises 78 genes divided into 13 groups (A-K, M, and S). Most of these bZIP transcription factors have been found to regulate plant growth, development, senescence, seed maturation, metabolic reactions, and stress responses [6]. Among the plant bZIP family, the formation of heterodimers between S_1_ (a subgroup of the S group) and C bZIP group members is referred to as the C/S_1_ bZIP network, which play an important role in the plant low-energy management system [7]. Under starvation conditions, SnRK1 phosphorylated *Arabidopsis* bZIP63 (group C) to enhance heterodimerization with particular S_1_ group members [8] and improved the transcriptional potential of S_1_ group members, such as Arabidopsis bZIP11 [3]. However, the transcriptional responses induced by darkness include the induction of stress-related and metabolic target genes by S_1_-bZIPs in SnRK1-dependent and -independent ways [8]. In addition, S_1_-bZIPs were found to be transcriptionally induced by abiotic stresses, leading to enhanced tolerance against cold, salt, and drought [9,10,11]. For example, the analysis of loss-of-function and gain-of-function mutations in Arabidopsis S_1_-bZIP member (AtbZIP1) suggested that AtbZIP1 regulates plant tolerance to abiotic stresses including salt, osmotic, and drought stresses [9]. Similarly, rice S_1_-bZIP member (OsbZIP71) overexpression led to enhanced tolerance to drought, salt, and osmotic stresses [12], suggesting that S_1_-bZIPs are an available resource to improve environmental stress tolerance, although the functional specificities of individual S_1_-bZIPs remain unknown.

Chinese cabbage (*Brassica rapa* L. ssp. pekinensis) is a vital cruciferous vegetable in Asia, particularly in China, Korea, and Japan. Its production yield is significantly affected by global climate change [13]. Although understanding the molecular mechanism of stress -tolerance modulation is essential, functional characterization of stress-induced genes in this plant remains a challenge. In a previous study, we identified 64 bZIPs as drought-induced differentially expressed genes. Among them, *Brassica rapa* S_1_-bZIP, known as BrbZIP-S, was induced by drought and salt stresses, indicating its possible involvement in abiotic stress responses [13]. To investigate the function of BrbZIP-S and evaluate its potential for engineering stress tolerance, we analyzed the impact of a heterologous gain-of-function approach of BrbZIP-S in *Nicotiana benthamiana*.

## 2. Materials and Methods

### 2.1. Plant Growth Conditions

Seeds of Chinese cabbage (*B*. *rapa* L. ssp. pekinensis) cultivar (Chunkwang) and two lines of *BrbZIP*-*S*-overexpressing *N*. *benthamiana* (OX3 and OX7; T3 generation) were germinated and grown in a growth chamber (16 h light and 8 h dark) at 24 °C. In addition, we chose regenerated plants, which survived on the selection medium but exhibited no transcription of *BrbZIP-S*, as transgenic control plants (TC). Six- to eight-week-old transgenic or TC plants were incubated at 45 °C for six hours or treated with dark stress for five days. Three biological replicates (ten plants for each replicate) in each treatment were carried out.

### 2.2. cDNA Synthesis and Quantitative Real-Time PCR (qRT-PCR) Analysis

Total RNA was isolated from the leaves of Chinese cabbage or *N*. *benthamiana* using FavorPrep Plant Total RNA Mini Kit (Favogen, Pingtung, Taiwan) and reverse-transcribed using the Toyobo cDNA synthesis kit (TOYOBO, Co., Ltd., Osaka, Japan). qRT-PCR was performed using the Toyobo SYBR-Green Master Mix. Specific primer pairs for each gene were used (Table S1) and the transcription levels of target genes were normalized to *NbEF1*.

### 2.3. Plasmid Construction, Plant Transformation, and Analysis of Subcellular Localization

Using Chinese cabbage cDNA as a template, the full-length BrbZIP-S (accession number: XM_009140057) was cloned into the gateway binary vector pGWB505 for the expression of BrbZIP-S. The binary vector was introduced into *Agrobacterium tumefaciens* GV3101 and used to transform *N*. *benthamiana*. Leaf disk explants of 3-week-old plants were inoculated into the Agrobacterium suspension, and co-cultivated in dark condition. After 48 h, leaf disks were selected with selection medium (MS medium containing 1 mg/L 6-benzylaminopurine, 0.1 mg/L indole-3-acetic acid, 300 mg/L cefotaxime, 30 mg/L hygromycin, 30 g/L sucrose, and 7.5 g/L plant agar). The transgenic lines showing hygromycin resistance were transplanted in soil and lines with a high level of BrbZIP-S-GFP protein were selected by RT-PCR.

GFP fluorescence was visualized using an Olympus FV1000 laser scanning confocal microscope (Tokyo, Japan). The DNA-binding reagent, 4′,6-diamidino-2-phenylindole (DAPI; Sigma-Aldrich, St. Louis, MO, USA), was used for staining nuclei.

### 2.4. Analysis of Proline and Pyrroline-5-Carboxylate (P5C) Contents

Leaves of *N*. *benthamiana* were homogenized in 3% sulfosalicylic acid. After centrifugation, the supernatant was used for the analysis of proline and P5C contents. The content of proline was quantified using a colorimetric assay as described by Eom et al. [14] and expressed as ng/mg of fresh weight (F.W.).

For analysis of P5C content, the supernatant of each sample was mixed with 10 mM of 2-aminobenzaldehyde, as described by La et al. [15]. After incubation at 37 °C for two hours, the absorbance at 440 nm was determined, calculated by using an extinction coefficient of 2.58 mM^−1^ cm^−1^, and expressed as nM/mg of F.W.

### 2.5. Determination of Invertase Activity and Sucrose, Glucose, and Fructose Contents

To determine the activity of invertase, 0.5 g of leaf grinding material was mixed in 1 mL extraction buffer (200 mM HEPES, 3 mM MgCl_2_, 1 mM EDTA, 2% glycerol, 0.1 mM PMSF, and 1 mM benzamidine) as described by Bonfig et al. [16]. After centrifugation, the supernatant was used to analyze the vacuolar invertase (NbV-inv) activity. The pellet was washed three times with distilled water, resuspended in the extraction buffer with 1 M NaCl, and subjected to another round of centrifugation. The supernatant was used to analyze the activity of extracellular invertase (NbC-inv). The activity of NbV-inv and NbC-inv was analyzed using GOD-POD reagent [16] and the absorbance at 595 nm was determined.

The contents of glucose, fructose, and sucrose were determined using the Sucrose/D-Fructose/D-Glucose Assay Kit (Megazyme, Wicklow, Ireland).

### 2.6. Determination of Chlorophyll (Chl), H_2_O_2_ Accumulation, Malondialdehyde (MDA) Content, and Protein Carbonyl Content

Ten milligrams of samples were extracted in 95% ethanol at 80 °C for 30 min, and an aliquot of the extracts was used to determine the chlorophyll (Chl) content. The contents of Chl a, b, and Chl a+b were calculated according to Czyczyło-Mysza et al. [17].

The accumulation of H_2_O_2_ in *N*. *benthamiana* leaves was carried out using the 3,3-diaminobenzidine (DAB) staining method, as described by Ji et al. [18]. In addition, MDA content was analyzed as described by Eom et al. [13] and expressed as nmol/mg of F.W. using an absorbance coefficient of 155 mM^−1^ cm^−1^.

To determine the protein carbonyl content, protein from N. benthamiana leaves was extracted using an extraction buffer, as described by Eom et al. [13]. The same concentrations of protein were used for analyzing content of protein carbonyl using a fluorometric protein carbonyl content assay kit (BioVision, Milpitas, CA, USA).

## 3. Results and Discussion

### 3.1. Sucrose-Induced Repression of Translation in BrbZIP-S

**Figure 1:**
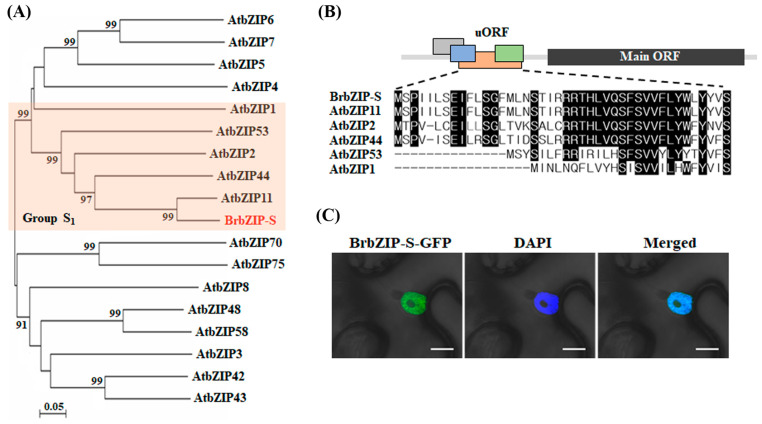
Characterization of *Brassica rapa* bZIP-S (BrbZIP-S). (**A**) Phylogenetic analysis between *Arabidopsis* S bZIPs and BrbZIP-S. (**B**) Evolutionary conserved uORF in BrbZIP-S. The predicted amino acid sequence encoded by SC-uORF of BrbZIP-S was aligned with *Arabidopsis* S_1_-bZIPs. (**C**) Subcellular localization of BrbZIP-S in *N. benthamiana* plants. Co-localization of GFP with DAPI confirms the localization of BrbZIP-S in the nucleus. Scale bar = 10 μm.

Among the plant bZIP family, the S group is the largest bZIP subfamily and is divided into three subgroups (S_1_, S_2_, and S_3_) [19]. As shown in Figure 1A, BrbZIP-S is phylogenetically closely related to the *Arabidopsis* S_1_-bZIP subgroup, which includes AtbZIP1, AtbZIP2, AtbZIP11, AtbZIP44, and AtbZIP53 [19]. Sucrose is a signaling molecule that negatively controls the translation of the S_1_-bZIP subgroup [20,21]. This sucrose-induced repression of translation (SIRT) is mediated by the presence of a highly conserved upstream open reading frame (uORF) found in the 5′ leader region of *S_1_-bZIP* transcripts, called the sucrose-controlled uORF (SC-uORF) [20,21]. *BrbZIP-S* contained four uORFs in its 5′ leader of which the third uORF showed high homology to the SC-uORF of *Arabidopsis S_1_-bZIPs* (Figure 1B), indicating that *BrbZIP-S* retains a SIRT mechanism mediated by a conserved SC-uORF.

Similar to other plant S_1_-bZIPs [22], BrbZIP-S-cGFP was localized in the nucleus (Figure 1C), indicating that BrbZIP-S is a nuclear protein. Although the basic region of bZIPs serves as a nuclear localization signal, phospho-mimicking mutations can disrupt the correct localization of bZIPs, indicating that phosphorylation regulates the subcellular localization of bZIP proteins, targeting either nuclear import or cytoplasmic retention [23,24]. However, the nuclear localization of AtbZIP53-cGFP was not changed by mimicking phosphorylation of conserved serines in the DNA-binding domain, suggesting that the functionality of the nuclear localization signal in S_1_-bZIPs is not disrupted by phosphorylation events [24].

### 3.2. BrbZIP-S Affects Sucrose Metabolism

**Figure 2:**
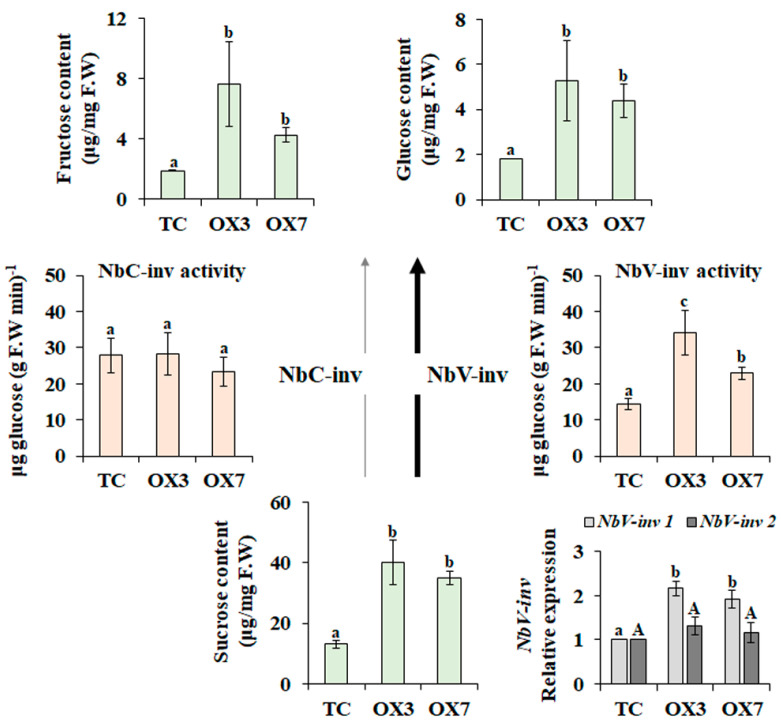
Sucrose metabolism in *BrbZIP-S*-overexpressing *N*. *benthamiana* plants (OX3 and OX7 lines). Total content of sucrose, glucose, and fructose was determined. The activities of vacuolar invertase (NbV-inv) and extracellular invertase (NbC-inv) were determined. Normalized expression levels of *NbV-inv 1* and *2* were expressed as relative to transgenic control (TC) plants. Means (±SE) with different letters are significantly different, according to Duncan’s multiple range test (<0.05).

Sucrose, the major form of carbon in higher plants, is used for internal regulation and physiological responses. As described above, S_1_-bZIPs, including BrbZIP-S, are known to be repressed by sucrose through translational inhibition [25,26], whereas overexpression of *S_1_-bZIP* increases the sugar content [26,27,28]. For example, fruit-specific expression of tomato *S_1_-bZIP* (*SlbZIP1*) or overexpression of strawberry *S_1_-bZIP* (strawberry *bZIP11*) resulted in tomato sweetening via the accumulation of sugar (sucrose, glucose, and fructose) [27,28]. Similarly, we found that the heterologous overexpression of *BrbZIP-S* induced sugar accumulation in *N*. *benthamiana* plants. As shown in Figure 2, the sucrose content was approximately 2.7- to 3-fold higher in transgenic lines (OX3 and OX7) than in TC plants. The transgenic plants also had higher levels of glucose (2.3- to 4-fold) and fructose (2.4- to 2.9-fold). In addition, overexpression of *BrbZIP-S* resulted in increased activity of vacuolar invertase. Furthermore, *N*. *benthamiana vacuolar invertase 1* (*NbV-inv 1*) was upregulated in all transgenic lines (Figure 2), suggesting that BrbZIP-S affected carbohydrate partitioning via a mechanism that includes the regulation of *NbV-inv 1* expression. These results suggest that BrbZIP-S is a potential gene for improving sweetness via the reprogramming of sugar metabolism.

### 3.3. Altered Proline Metabolism in BrbZIP-S Transgenic N. benthamiana Plants

**Figure 3:**
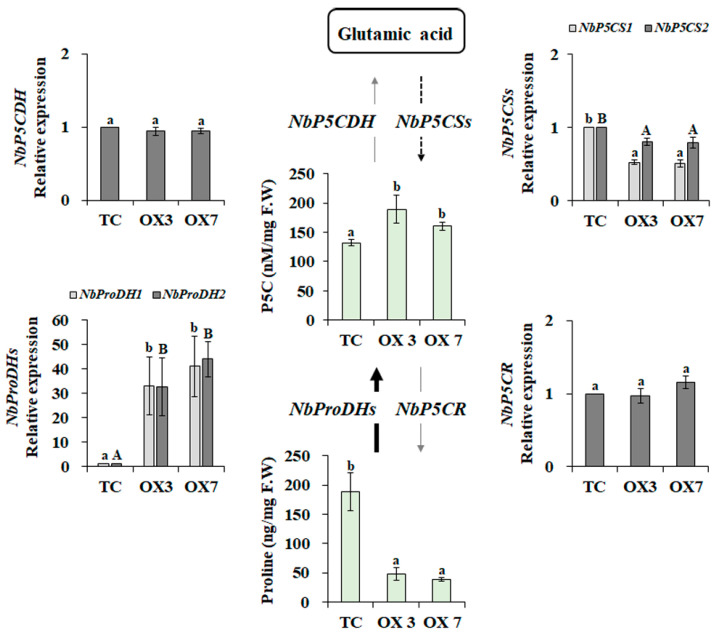
Proline metabolism in *BrbZIP-S* overexpressing *N*. *benthamiana* plants (OX3 and OX7 lines). Proline and pyrroline-5-carboxylate (P5C) contents were determined in *BrbZIP-S*-overexpressing (OX3 and OX7) and transgenic control (TC) plants. Expression levels of *ProDHs* (proline dehydrogenases), *P5CDH* (delta-1-pyrroline-5-carboxylate dehydrogenase), *P5CR* (pyrroline-5-carboxylate reductase), and *P5CSs* (pyrroline-5-carboxylate synthetases) were expressed as relative to TC plants. Means (±SE) with different letters are significantly different (Duncan’s multiple range test, *p* < 0.05).

One of the most important multifunctional amino acids in plants is proline, which is a proteinogenic amino acid synthesized from glutamate and ornithine [29]. Proline biosynthesis is induced by various stressors, and its catabolism is activated in darkness and during stress relief via TOR- and SnRK1-dependent signaling [30,31]. Overexpression of *AtbZIP11* has been shown to lead to reduced proline content [32], indicating that S_1_-bZIP plays a role in coordinating sucrose and proline metabolism. We showed that OX plants contained a much lower proline level than the TC plants (Figure 3). In addition, P5C was slightly increased in OX plants, indicating that proline catabolism was activated by BrbZIP-S.

In higher plants, proline catabolism occurs in the mitochondria by the catalytic action of two enzymes, proline dehydrogenase (ProDH; EC 1.5.99.8), which produces pyrroline-5-carboxylate (P5C) from proline, and delta-1-pyrroline-5-carboxylate dehydrogenase (P5CDH; EC 1.5.1.12), which converts P5C to glutamate. The two enzymes pyrroline-5-carboxylate synthetase (P5CS; EC not assigned) and pyrroline-5-carboxylate reductase (P5CR; EC 1.5.1.2) are involved in proline biosynthesis catalyzing glutamate to proline [29,31]. In *Arabidopsis*, S_1_-bZIPs, including bZIP2, bZIP11, bZIP44, and bZIP53, function as transcriptional activators of *ProDH* genes [33], and the formation of the C/S_1_ bZIP network is required for controlling *ProDH* expression [11]. Here we showed that *N*. *benthamiana ProDH* (*NbProDH*) *1* and *2* were expressed at higher levels in OX plants compared to TC plants (Figure 3). In contrast, the expression levels of *NbP5CS 1* and *2* were down-regulated (Figure 3). These results suggest that BrbZIP-S induces metabolic changes in proline metabolism.

### 3.4. BrbZIP-S Controls Darkness-Induced Senescence in Transgenic Plants

**Figure 4:**
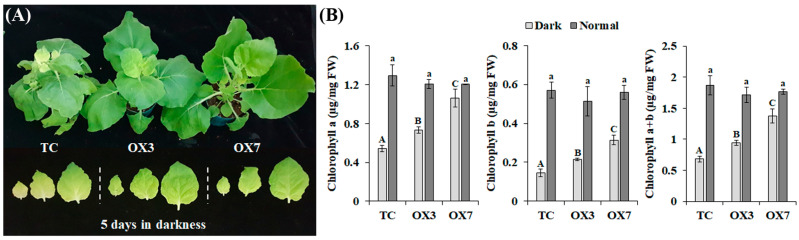
*BrbZIP-S* overexpression delayed darkness-induced senescence in *N*. *benthamiana* plants. (**A**) The phenotype of *BrbZIP-S*-overexpressing plants (OX3 and OX7 lines) and transgenic control (TC) plants after five days in darkness. (**B**) Chlorophyll (Chl) contents of plants were determined. Uppercase denotes significance between the Chl levels under darkness, whereas lower-case denotes significance between the Chl levels under normal conditions (Duncan’s multiple range test, *p* < 0.05).

Various investigations have shown that unfavorable environmental stressors, including light deprivation, lead to rapid leaf senescence [34]. The understanding of darkness- or age-induced senescence is of high economic relevance as senescence can significantly decrease the post-harvest shelf-life of a plant as well as lead to yield loss in agriculture [35]. In *Arabidopsis*, the low content of trehalose 6-phosphate, a non-competitive inhibitor of SnRK1 [36], was shown to lead to delayed senescence [37]. During darkness-induced senescence, it was found that ProDH-mediated proline catabolism provides energy and glutamate, which play a crucial role in nitrogen remobilization [38]. Therefore, we presumed that the increasing proline catabolism by BrbZIP-S delays darkness-induced senescence. To test this hypothesis, we transferred six-week-old plants to extended darkness for five days and then analyzed the Chl levels, an integral part of leaf senescence [39]. As shown in Figure 4, the levels of Chl a, Chl b, and Chl a+b decreased by 58.2% (1.29 µg/mg F.W. to 0.54 µg/mg F.W.), 74.7% (0.57 g/mg F.W. to 0.14 g/mg F.W.), and 63.3% (1.87 µg/mg F.W. to 0.68 µg/mg F.W.), respectively, in plants kept in darkness, compared to those in normal growth conditions. However, the contents of Chl a, Chl b, and Chl a + b were significantly higher in OX3 and OX7 lines than in TC plants under dark conditions, indicating that BrbZIP-S overexpression delayed Chl degradation during darkness-induced senescence. It was previously shown that nutrient-deprivation-induced senescence and age-dependent senescence were delayed in plants overexpressing *KIN10* (the main component of Arabidopsis SnRK1) and the rice *SnRK1*, respectively [3,40], suggesting the essential role of S_1_-bZIPs, including BrbZIP-S, in plant survival is through inducing remobilization of proline under darkness and nutrient deprivation conditions.

### 3.5. Overexpression of BrbZIP-S Increases the Heat Tolerance of N. benthamiana

**Figure 5:**
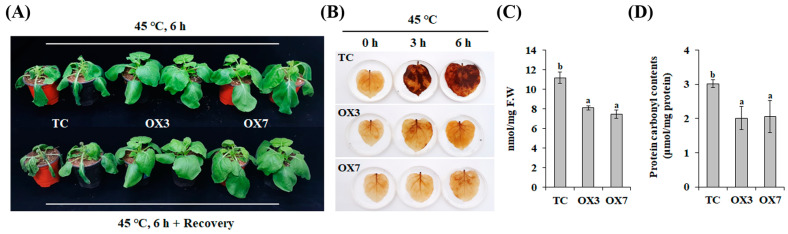
*BrbZIP-S* overexpression improved heat tolerance in *N*. *benthamiana* plants. (**A**) The phenotype of *BrbZIP*-*S*-overexpressing plants (OX3 and OX7 lines) and transgenic control (TC) plants after exposure to 45 °C for six hours and recovery overnight at normal growth conditions. Changes in H_2_O_2_ level (**B**), malondialdehyde (MDA) level (**C**), and protein carbonyl content (**D**) were determined. Means (±SE) with different letters are significantly different (Duncan’s multiple range test, *p* < 0.05).

Among unfavorable environmental conditions, higher temperatures above critical thresholds affect crop growth and development, leading to significant yield loss. Therefore, improving heat tolerance is of profound importance to the production yields of crops and will greatly secure food security [41]. Plant S_1_-bZIPs play a key role in plant innate immunity and the response to unfavorable environmental conditions [11]. In our previous study, *BrbZIP-S* was induced under abiotic stress conditions [13], indicating the possible involvement of BrbZIP-S in the stress response. To analyze the function of BrbZIP-S in heat stress responses, OX and TC plants were treated at 45 °C for six hours. As shown in Figure 5A, the leaves of the OX3 and OX7 lines were less wilted than those of the control plants. In addition, H_2_O_2_ was less accumulated in the OX3 and OX7 lines than in the TC plants (Figure 5B). After the subsequent recovery incubation at 25 °C, severe damage was observed in the TC plants, whereas only a slight wilting was seen in the OX3 and OX7 lines (Figure 5A). The levels of MDA and protein carbonyls were significantly lower in OX lines than in TC plants (Figure 5C,D), indicating that BrbZIP-S increased heat tolerance in *N*. *benthamiana* plants.

Soluble sugars are osmoprotectants, which can protect cell membranes by scavenging toxic reactive oxygen species generated under various stress conditions, including heat stress [42,43]. In addition, SlbZIP1 and OsbZIP71 exert important roles in abiotic stress tolerance via modulating abscisic acid (ABA)-mediated pathways [12,44]. Under heat stress conditions, the transcription levels of genes involved in sucrose metabolism including sucrose synthase and invertase, were increased by ABA [45]. Therefore, one possible explanation should be that the BrbZIP-S-induced sugar metabolism reprogramming seen above (Figure 2) contributes to improved heat tolerance.

## 4. Conclusions

Energy homeostasis under energy-deprived conditions is very common and important in regulating plant defense responses. In this study, we analyzed the physiological function BrbZIP-S, and suggested that BrbZIP-S acts as a positive factor in stress tolerance against darkness and heat stress. BrbZIP-S may be regulated by stress response pathways via a complex network mediated by ABA, sugar, and proline. Further functional dissection of S_1_-bZIP proteins, their targets, and their interplay in signaling pathways mediated by energy homeostasis will contribute to our understanding of plant responses under environmental stress conditions.

## Data Availability

The data presented in this study are available on request from the corresponding author. The data are not publicly available due to reasons of privacy.

## Supplementary Materials

The following supporting information can be downloaded at: https://www.mdpi.com/article/10.3390/biology12040517/s1, Table S1: Primer sequences for cloning and qPCR analysis.
