# Supplementary material for: Overexpression of the Brassica rapa bZIP Transcription Factor, BrbZIP-S, Increases the Stress Tolerance in Nicotiana benthamiana
Source: Biology (Basel). 2023 Mar 29;12(4):517. doi: 10.3390/biology12040517 (PMC10136179; doi:10.3390/biology12040517)
Supplement: Supplementary file 1 [file biology-12-00517-s001.zip › biology-2281469-supplementary.pdf]

biology

MDPI

Supplementary Materials

Overexpression of the *Brassica rapa* bZIP Transcription Factor, *BrbZIP-S*, Increases the Stress Tolerance in *Nicotiana benthamiana*

Table S1. Primer sequences for cloning and qPCR analysis.

	Name	Sequences
Full-length cloning	BrbZIP-S-F	CACCATGGACTCGTCGTCGTC
	BrbZIP-S-R	ATACAGTAACGCATCAGAAGA
qPCR analysis	NbP5CS1-F	ATCTTGATGGCAAGGCTTGTGCTG
	NbP5CS1-R	AAGCTGAGCTGAGGTTACGTCCAA
	NbP5CS2-F	CCTGTTCTTGGTCATGCTGATGGT
	NbP5CS2-R	GCATTGCAGGCTGCTGGATAATCA
	NbP5CR-F	TGGAGAAGGCTGGATTTCGTGGTA
	NbP5CR-R	TTCCGGCTGGTCCACCACTTA
	NbProDH1-F	AGCTATGTGCGTAGCCTCTT
	NbProDH1-R	AGGTGCTACAACAGATGGAGAAAG
	NbProDH2-F	GGTCTATTATATTGTAGCACCTTTGT
	NbProDH2-R	CATTTCGGCTCCACTTTGGGTA
	NbP5CDH-F	TCCAAGTGCCCGAAACATGGTCTA
	NbP5CDH-R	GAAACCTCAGGCAAGCCAAGCATA
	NbV-inv 1-F	GCCACCCACCATTTCCATTT
	NbV-inv 1-R	GGAGAGAAGAATGCCGGAGA
	NbV-inv 2-F	TGAAGTGGACAAAGCAGCAC
	NbV-inv 2-R	CTCGGCCATCAGTTCCTTTG
	NbEF1-F	CACGCATTGCTTGCTTTCA
	NbEF1-R	TCCATCTTGTTACAGCAGCAAATC
